# Canine IL4-10 fusion protein provides disease modifying activity in a canine model of OA; an exploratory study

**DOI:** 10.1371/journal.pone.0219587

**Published:** 2019-07-11

**Authors:** E. M. van Helvoort, J. Popov-Celeketic, N. Eijkelkamp, K. Coeleveld, M. A. Tryfonidou, C. D. Wijne, C. E. Hack, F. P. J. G. Lafeber, S. C. Mastbergen

**Affiliations:** 1 Department of Rheumatology & Clinical Immunology, University Medical Center Utrecht, Utrecht University, Utrecht, Netherlands; 2 Laboratory of Translational Immunology, University Medical Center Utrecht, Utrecht University, Utrecht, Netherlands; 3 Laboratory of Neuroimmunology and Developmental Origins of Disease, University Medical Center Utrecht, Utrecht University, Utrecht, Netherlands; 4 Department of Clinical Sciences of Companion Animals, Faculty of Veterinary Medicine, Utrecht University, Utrecht, Netherlands; Chang Gung University, TAIWAN

## Abstract

**Objective:**

An ideal disease modifying osteoarthritis drug (DMOAD) has chondroprotective, anti-inflammatory, and analgesic effects. This study describes the production and characterization of a canine IL4-10 fusion protein (IL4-10 FP) and evaluates its *in vivo* DMOAD activity in a canine model of osteoarthritis (OA).

**Design:**

The canine Groove model was used as an *in vivo* model of degenerative knee OA. Six weeks after OA induction dogs were intra-articularly injected weekly, for ten weeks, with either IL4-10 FP or phosphate buffered saline (PBS). In addition to the use of human IL4-10 FP, canine IL4-10 FP was developed and characterized *in vitro*, and tested *in vivo*. Force plate analysis (FPA) was performed to analyze joint loading as a proxy measure for pain. After ten weeks dogs were euthanized and cartilage and synovial tissue samples were analyzed by histochemistry (OARSI scores) and biochemistry (cartilage proteoglycan turnover).

**Results:**

Repetitive intra-articular injections with human IL4-10 FP led to antibody formation, that blocked its functional activity. Therefore, a canine IL4-10 FP was developed, which completely inhibited LPS-induced TNFα production by canine blood cells, and increased proteoglycan synthesis of canine cartilage *in vitro* (p = 0.043). *In vivo*, canine IL4-10 FP restored the, by OA impaired, joint loading (p = 0.002) and increased cartilage proteoglycan content (p = 0.029).

**Conclusions:**

This first study on the potential DMOAD activity upon prolonged repeated treatment with IL4-10 FP demonstrates that a species-specific variant has anti-inflammatory and chondroprotective effects *in vitro* and chondroprotective and analgesic effects *in vivo*. These data warrant further research on the DMOAD potential of the IL4-10 FP.

## Introduction

Osteoarthritis (OA) is characterized by changes in all (peri-)articular tissues, such as cartilage, (subchondral) bone, synovial tissue, ligaments, and muscles[1, 2]. Currently, OA treatment is mostly symptomatic and mainly focused on pain reduction. If conservative treatments (e.g. physical therapy), analgesic drugs (e.g. acetaminophen), and/or anti-inflammatory drugs (e.g. non-steroidal anti-inflammatory drugs) fail, limited joint preserving surgical options are available (e.g. joint distraction), and finally a total joint replacement becomes inevitable[2]. Therefore, there is still a great need for joint preserving conservative drug treatments. Such treatments should ideally be chondroprotective (regenerative), anti-inflammatory, and analgesic, and should be preferably combined in one drug. At present such treatments do not exist, although multiple drugs are studied as potential disease modifying OA drug (DMOAD). Intra-articular (i.a.) administration of fibroblast growth factor-18 (FGF18) has demonstrated chondroprotective effects in a bovine *in vitro* model[3], in an *in vivo* rat model[4] and in a randomized, double-blind, placebo-controlled trial, although pain relief was not achieved[5, 6]. Transforming growth factor-beta1 (TGF-β1) producing human chondrocytes were able to generate cartilage with hyaline-like characteristics in two different animal models[7] and demonstrated clinical benefits in multiple clinical trials[8–12] with small, not statistically significant but encouraging chondroprotective activity[8, 12, 13], and anti-inflammatory activity[13]. SM04690, a Wnt pathway inhibitor, induced chondrocyte differentiation, reduced cartilage catabolism *in vitro*, and showed chondroprotective activity in a rat model of OA[14], and in humans[15]. None of these studies demonstrated chondroprotective, anti-inflammatory, and analgesic effects in combination.

Interleukin 4 (IL4) and IL10, two anti-inflammatory cytokines, are suggested to have therapeutic potential in OA: synovial cells and chondrocytes express IL4 and IL10 receptors (IL4R, IL10R)[16–18]. IL4R signaling changes mechano-transduction in chondrocytes linked to matrix turnover in OA[19] and variants in the IL4R alpha gene are associated with susceptibility to osteoarthritis[20]. Besides, IL10 and IL4 inhibit chondrocyte apoptosis and cartilage breakdown[21, 22] and reduce synovial inflammation by reversing prostaglandin E_2_ production by OA synovial fibroblasts[23].

IL4 and IL10 do have overlapping and complementary activities[24] and combined administration showed promising effects in experimental models of arthritis[25, 26]. Recently, we developed a human fusion protein of IL4 and IL10 (hIL4-10 FP)[27] that has DMOAD properties in multiple models of osteoarthritis[28]. Human OA cartilage tissue explants were demonstrated to express elevated levels of IL4R and IL10R, rendering OA cartilage more sensitive to the IL4-10 FP.

Moreover, two i.a. injections of IL4-10 FP in dogs with experimental OA were analgesic and *in vitro* IL4-10 provided chondroprotective and anti-inflammatory activity in human cartilage and synovial tissue explants. These findings warranted further *in vivo* evaluation on the potential disease modifying properties of this novel IL4-10 fusion molecule. As such we designed an *in vivo* study using the canine Groove model of osteoarthritis to study the chondroprotective, anti-inflammatory, and analgesic activity of the IL4-10 FP upon repeated i.a. injections.

## Material and methods

### Production and characterization of IL4-10 FP

hIL4-10 FP was produced as previously published[29]. Canine IL4-10 FP (cIL4-10 FP) was produced by transient transfection of HEK293F cells with pcDNA3.1-neo expression vector (Invitrogen; Carlsbad, CA) with dual CMV promotor. The vector contained two transgenes: cDNA coding for cIL4-10 FP and cDNA coding beta-galactoside-2, 3-sialyl-transferase to optimize glycan capping with sialic acid. To enable purification, a hexa-histidine affinity tag was cloned at the N-terminus of cIL4-10 FP. Cells were cultured in GIBCO FreeStyle 293 Expression Medium (Invitrogen; Carlsbad, CA). Cells were subcultured three to four times prior to transfection and transfected with 293fectin (Invitrogen; Carlsbad, CA) at the cell viability of at least 90%.

The cIL4-10 FP was purified using Ni-NTA agarose (Qiagen; Hilden) according to manufacturer protocol. Purified canine protein was dialyzed overnight against 2L of phosphate buffered saline (PBS; pH = 7.4), sterile filtered and stored at -80°C. Purity of cIL4-10 FP batches was evaluated by Coomassie-stained 12% SDS-PAGE gel and HP-SEC analysis.

For SDS-PAGE samples were diluted 1:1 in 2x Leammli Sample Buffer (Bio-Rad; Hercules, CA) containing 100mM dithiotreitol (Sigma-Aldrich; Saint Louis, MO), incubated ten minutes at 95°C, and loaded on a 12% polyacrylamide gel (Mini-PROTEAN-TGX; Bio-Rad; Hercules, CA). Electrophoresis was performed at 150V for 1,5 hours, under reducing conditions (Tris/glycine/SDS buffer; Bio-Rad; Hercules, CA). Protein bands were visualized by InstantBlue protein stain (Expedeon; Cambridge).

After electrophoresis, proteins were transferred to a nitrocellulose membrane (Trans-Blot Turbo system, Bio-Rad; Hercules, CA). Membranes were blocked in 5% milk (Elk; Campina; Zaltbommel) in PBST (phosphate buffer saline with 0.1% Tween-20, Merck; Darmstadt) and incubated overnight with a monoclonal mouse anti-canine IL4 or monoclonal mouse anti-canine IL10 (R&D systems; Minneapolis, MN) in PBST containing 1% milk. Membranes were subsequently incubated with goat anti-mouse IgG-HRP (Santa Cruz Biotechnology; Dallas, TX) for one hour at room temperature. To visualize the bands ECL Western Blotting Substrate was added according to manufacturer protocol (Pierce, Thermo Fisher Scientific; Waltham, MA).

### Bioactivity check of cIL4-10 FP in vitro

To assess biological activity of the cIL4-10 FP two *in vitro* experiments were performed, before *in vivo* testing. To evaluate anti-inflammatory activity of cIL4-10 FP, heparinized canine blood was diluted 1:10 in RPMI1640 medium (Gibco, Thermo Fisher Scientific; Waltham, MA) supplemented with 1% penicillin/streptomycin (Gibco, Thermo Fisher Scientific; Waltham, MA), and incubated with lipopolysaccharide (LPS; Sigma-Aldrich; Saint Louis, MO) at 100ng/ml and cIL4-10 FP, recombinant canine IL4 (R&D systems; Minneapolis, MN) or recombinant canine IL10 (R&D systems; Minneapolis, MN), both as controls, at 0.001-3nM for 18 hours at 37°C, 5% CO_2_. Culture supernatants were then assayed for canine Tumor Necrosis Factor alpha (TNFα; R&D systems; Minneapolis, MN) using Enzyme linked immuno sorbet assay (ELISA). The inhibition of TNFα production (by cIL4-10 FP, cIL4 or cIL10) was calculated according to the formula: Inhibition (%) = (1-(A-B)/(C-B)) x 100, where A = TNFα levels in LPS-stimulated cultures treated with cIL4-10 FP, cIL4 or cIL10; B = TNFα levels in unstimulated culture; and C = TNFα levels in LPS-stimulated culture.

To assess chondroprotective activity *in vitro*, surplus cartilage of five healthy dogs used for unrelated studies was used to determine cartilage proteoglycan (PG) synthesis. Per dog, 16 cartilage explants were harvested and cultured in a 96-wells plate (Thermo Fisher Scientific; Waltham, MA) in culture medium (Gibco, Thermo Fisher Scientific; Waltham, MA) containing TNFα (10ng/mL) to impair chondrocyte activity with or without cIL4-10 FP (100ng/mL) (n = 8 for each condition). Samples were cultured for four days at 37°C and chondrocyte PG synthesis was determined as described before[30]. In short, after one hour of pre-culture, 20 μCI Na_2_^35^SO_4_ (Dupont, NEX-041-H, carrier free; Wilmington, DE) in DMEM was added to each sample. After four hours of labeling, samples were washed with PBS and digested with papain for two hours at 65°C. Glycosaminoglycans (GAGs) were precipitated by addition of cetylpyridium chloride (CPC). ^35^SO_4_^2—^labelled GAGs were measured by liquid scintillation on a TRI-CARB 2800TR (PerkinElmer; Waltham, MA). Values were normalized to specific activity of the pulse medium, pulse time, and wet weight of the explants and expressed as nmoles of sulphate incorporated per hour per gram wet weight of the cartilage (nmol.h^-1^.g^-1^). Data of eight samples were averaged for each cartilage donor (dog).

### In vivo canine model of OA

The study was approved by the animal ethical committee of the Utrecht University (DEC 2014.III.09.081). For logistical reasons the experiment was split in two sets of eight dogs (four treated with IL4-10 FP and four with PBS).

Skeletally matured female mixed breed dogs (46 ± 31 months, 30.4 ± 17.4 kg; Marshall BioResources; North Rose, NY) were matched on weight and walking pattern and then split into two comparable groups. Dogs were housed in pairs and let out on a patio in large groups for at least 2 hours a day. They were fed a standard diet and had water *ad libitum*. OA was induced in the right leg according to the Groove model[31]. Dexdomitor (0.02 mg/kg i.m.) and Ketamin (0.5 mg/kg i.m.) were used as pre-medication before induction with thiopental (20mg/kg i.v.). Anesthesia was done with Sufentanil (0.0225 mg/kg/h i.m.) and Isofluran. Six to ten grooves of 2-3mm depth were made in the femoral cartilage as described in detail previously. Dogs were treated with painkillers (Carprofen; 4mg/kg p.o.) and antibiotics (Cefazolin; 35mg/kg i.v. pre-surgery and Synulox; 15mg/kg p.o. twice a day post-surgery) one day before surgery and during five days after surgery. During the first week after surgery regular wound checks were performed. Starting 2 days after surgery dogs were let out on the patio again. General control of animals and housing were performed during the whole study period. Based on previous experiments it was not expected that clinical symptoms (e.g. infection due to OA induction) would be of such degree that the human endpoint would be reached and euthanasia would be inevitable.

The Groove model is known to be an intrinsic cartilage degenerative model demonstrating progressive cartilage degradation[30, 32]. The model has been specifically designed to test chondroprotective treatments because treatment is not counteracted by a permanent trigger (like joint instability) or by a dominating inflammatory component (like chemically induced models). As such this model is explicitly suitable for testing DMOAD activity but not mainly anti-inflammatory activity. After six weeks of OA development, dogs were treated weekly with i.a. injections in their affected knee for ten successive weeks. Dogs in the treatment group were injected with IL4-10 FP (10μg in 500μL) whilst dogs in in the control group were injected with PBS (500μL). The left contralateral knee of each dog served as an internal control. Periodically during treatment blood was drawn and force plate analyses were performed providing data on joint loading as a proxy for pain. After the treatment period of ten weeks, dogs were euthanized and cartilage and synovial tissue samples were harvested. Natriumpentobarbital i.v. was used as euthesate. Ten minutes before euthanasia, dogs were injected with Dexdomitor (0.02 mg/kg i.m.) and Ketamin 0.5 mg/kg i.m.)

### Experimental set-up

The first part of the canine *in vivo* experiment was performed treating four dogs i.a. with human IL4-10 FP and four dogs with i.a. PBS as a control group.

**Fig 1:**
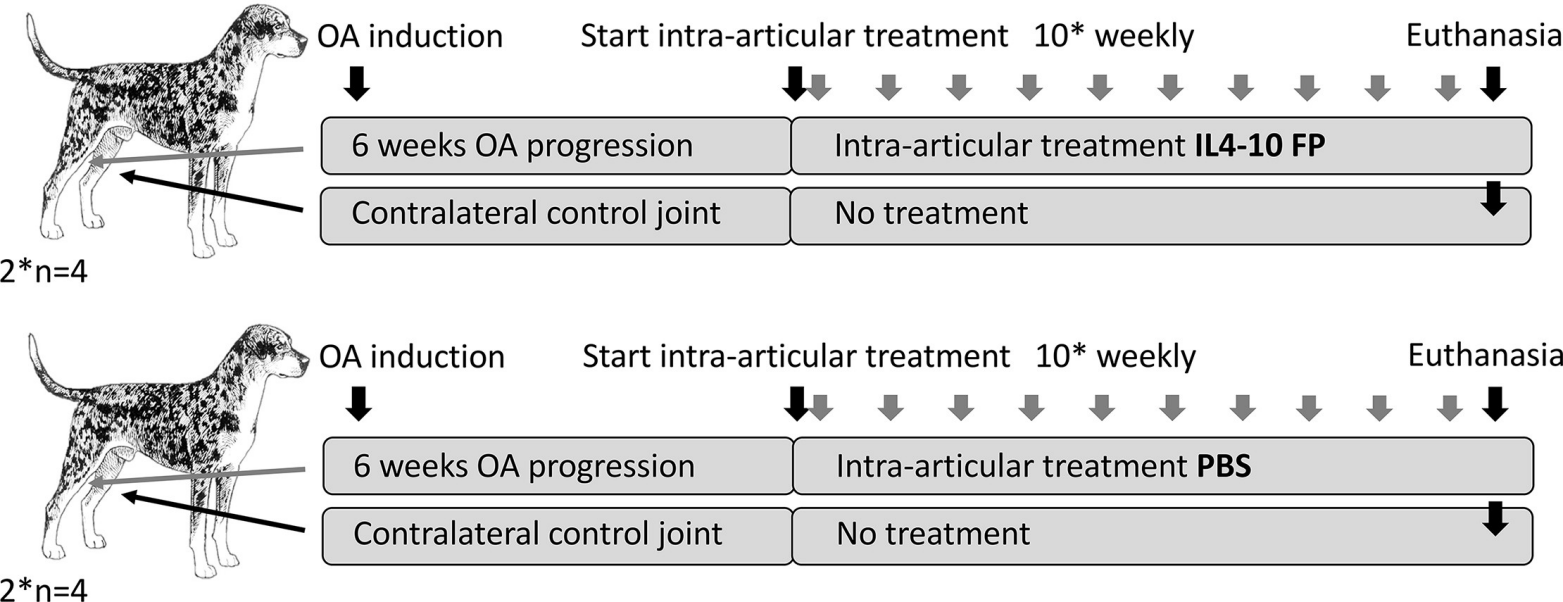
Experimental set-up of the canine *in vivo* experiment. The first part of the canine *in vivo* experiment was performed treating four dogs with i.a. human IL4-10 FP and four dogs with i.a. PBS as a control group. Because of IL4-10 FP neutralizing antibody formation the second part was performed treating four dogs with i.a. canine IL4-10 FP and four dogs with i.a. PBS.

As human IL4-10 FP turned out to be immunogenic in dogs after multiple i.a. injections, the canine IL4-10 FP was produced, characterized and tested for *in vitro* bioactivity as described above. Subsequently, the second part of the canine *in vivo* experiment was performed using this canine IL4-10 FP, treating four dogs i.a. with canine IL4-10 FP and four with i.a. PBS as a control group (Fig 1)

### Immunogenicity assessment for IL4-10 FP

ELISA was used to determine Immunoglobulin G (IgG) titers in serum of injected dogs. Serum was drawn before start of treatment and after every i.a. injection. In case of antibody detection after the tenth injection, intermediate time points were analyzed as well to identify when antibody formation was initiated.

After demonstrating the formation of Immunoglobulin G (IgG) in the hIL4-10 FP injected dogs, whole blood assays were performed to evaluate the neutralizing effects of these antibodies. Two 96-wells plate were coated with hIL4-10 FP. One plate was covered with dilutions of serum containing possible IgGs against hIL4-10 FP (after ten injections) and one plate was covered with dilutions of serum without IgGs against hIL4-10 FP (before ten injections). Subsequently TNFα inhibiting capacity of the hIL4-10 FP was determined.

### Force plate analysis

In order to evaluate pain, force plate analysis (FPA) was performed to determine joint (un)loading as a proxy for pain. Vertical peak force (Fz), braking force (Fy+), and propulsive force (Fy-) were determined. The ratio of the affected hind leg over the contralateral control hind leg was calculated for each dog for each time point. FPA was performed before Groove surgery to determine baseline values and during the treatment period before and 24 hours after the 1^st^, 6^th^ and 9^th^ injection.

### Assessment of synovial inflammation

Macroscopic scoring of synovial tissue samples was done on digital photographs of the synovial fat pad according to OARSI (range 0–5)[33]. Knee synovial tissue samples were embedded in Tissue Tek (Sakura; Torrance, CA) and stored at -80°C. Sections of 5μm were cut and stained with hematoxylin-eosin (Mayer’s hemalum solution; Merck, Darmstadt) and number of cell layers, lining characteristics and cell infiltration were scored from 0–6 (total score ranged from 0–18) according to OARSI[33]. Sections were analyzed by two observers blinded for treatment, and an average of six sections (three sections per observer) was calculated for each knee.

### Assessment of cartilage degeneration

Macroscopic scoring of cartilage tissue samples was done on digital photographs of the tibial plateau according to OARSI[33]. Also histologic cartilage degeneration was evaluated in tibial cartilage only, because grooves were applied in the femoral cartilage. Four samples of tibial cartilage were used. Cartilage samples were fixed in formalin and embedded in paraffin. Sections of 5 μm were cut and stained with Safranin-O-fast-green (Saf-O). Sections were scored for cartilage structure, chondrocyte pathology and PG staining from 0–12 (total score ranged from 0–36) according to OARSI[33]. All sections were stained in the same Safranin-O baths. Each sample (four per knee) was scored by two observers blinded for treatment. An average of eight scores was used for statistical evaluation. For one dog in the cIL4-10 FP group all four pieces of the right tibia could not be evaluated due to technical issues. Data was imputated by taking the mean change of the remaining three dogs and adding this value to the value of the contralateral knee.

### Cartilage proteoglycan (PG) turnover

The surgically untouched tibial cartilage was used for determining *ex vivo* PG turnover, as described before[30]. For determining PG content the glycosaminoglycans (GAGs) in a papain digest of the tissue samples were precipitated and stained with Alcian Blue dye solution[34]. This staining was quantified photometrically by change in absorbance at 620nm. Chondroitin sulphate (Sigma-Aldrich C4383; Saint Louis, MO) was used as a reference. Values were normalized to wet weight of the cartilage explants (mg/g). For total release of PGs, GAGs in the culture medium were precipitated from the medium, stained with Alcian blue dye solution and quantified photometrically with chondroitin sulphate as a reference. The total amount of GAGs released (blue staining) is expressed as a percentage of the original tissue content (% total PG release).

Determination of PG synthesis is described above (under *in vitro* bioactivity experiments). Release of newly formed PGs was determined as a measurement of retention of newly formed PGs. The release of ^35^SO_4_^2-^ -labelled PGs in the medium during three days was determined. After labelling, the samples were rinsed three times for 45 minutes in 1.5mL culture medium and then incubated in 200μL fresh culture medium without sulphate label. After three days released GAGs in the culture medium were precipitated by Alcian Blue dye solution. ^35^SO_4_^2-^ -labelled GAGs were measured by liquid scintillation analysis and values were normalized to wet weight of the explants. Release of newly formed PGs is normalized to total PG synthesis rate and expressed as percentage release of newly formed PGs (% release newly formed PGs).

An average of eight samples taken from predefined locations, identical for contralateral control and experimental joints, of the tibial cartilage was used as representative value for each joint.

Previous research shows that values of contralateral (left) knees are comparable to baseline values of experimental (right) knees.

### Statistical analysis

Statistical analyses were done using SPSS Statistics 25 (IBM; Armonk, NY) A Wilcoxon-signed rank test was used to analyze the *in vitro* bioactivity. For FPA, a linear mixed model, correcting for time, baseline values, and pre-injection values was used to evaluate the effects on joint loading 24 hours after i.a. injection. For inflammation and cartilage degeneration parameters mean differences between experimental knees and contralateral knees were determined and considered to represent the effect of OA induction and treatment with either IL4-10 FP or PBS within the experimental knees. The effects within the experimental knees were compared between PBS and IL4-10 FP group using Mann-Whitney U tests. P-values <0.05 were considered as statistically significant for all tests.

## Results

### Canine IL4-10 FP production and characterization

**Fig 2:**
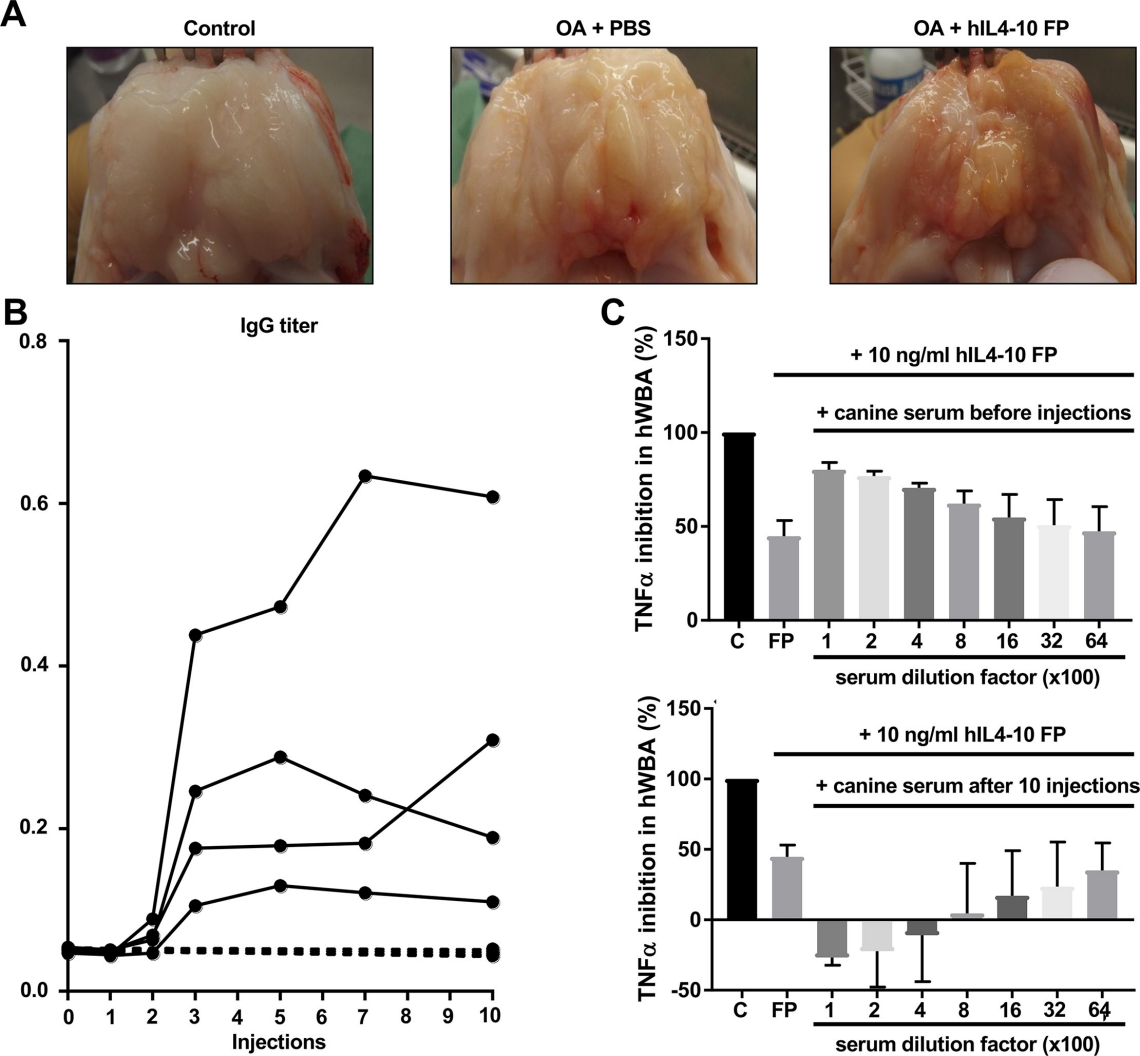
Immune response against hIL4-10 FP. A. Macroscopic pictures of synovial fatpad of control, PBS injected and hIL4-10 FP injected knees. B. IgG titers in serum of PBS injected dogs (dotted line) and hIL4-10FP injected dogs (continuous line). C. TNFα inhibition of hIL4-10 FP after adding serum taken before i.a. injections (above) and after i.a. injections with hIL4-10 FP (below). TNFα inhibition is expressed as percentage of inhibition in serum of non-injected dogs.

Human IL4-10 FP was immunogenic in dogs upon repeated i.a. injections, leading to the formation of antibodies after only three repetitive i.a. injections (Fig 2B). Whole blood assays evaluating the TNFα-inhibition of hIL4-10 FP after adding serum of hIL4-10 FP injected dogs after ten injections showed a complete loss of activity of the hIL4-10 FP, demonstrating a neutralizing effect of the IgGs (Fig 2C). Moreover, the immunogenicity caused severe synovial inflammation (Fig 2A), visible as diffuse discoloration due to neovascularization as well as tissue proliferation with villi formation.

**Fig 3:**
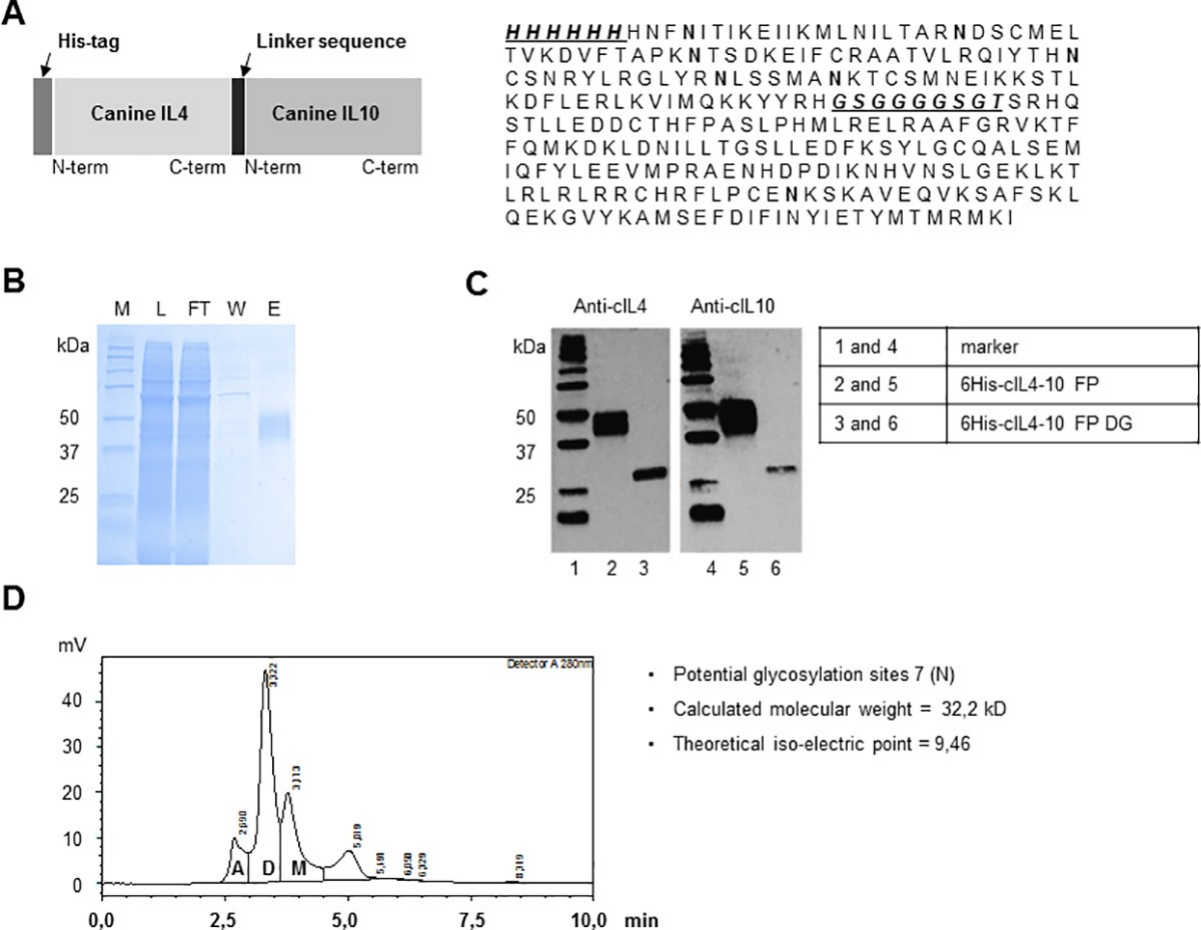
Molecular characterization of cIL4-10 FP. A. Schematic overview of the cIL4-10 FP. B. Coomassie Brilliant Blue stained SDS gel in Ni-NTA protein purification steps; M: Marker, L: Load, FT: Flow-through, W: Wash, E: Elution. C. Western blot analysis of purified cIL4-10 FP (untreated and deglycosylated; DG). D. HP-SEC profile showing; A: Aggregate, D: Dimer, M: Monomer.

Therefore, a canine variant of the IL4–10 FP was developed. In Fig 3A schematic representation is shown of the cIL4-10 FP and its amino acid sequence, including the linker sequence and seven predicted glycosylation sites. Purified cIL4-10 FP was detected on Coomassie stained SDS gel as a smear composed of multiple protein bands corresponding to a molecular mass of 37 – 50kDa (Fig 3B). A smeared protein band was also detected on a Western Blot by anti-IL4 and anti-IL10 antibodies, while after deglycosylation of cIL4-10 FP with PNGaseF only one sharp protein band of 30kDa was detected, as expected based on amino acid sequence (Fig 3C), indicating the apparent smear represented different glycoforms of cIL4-10 FP. HP-SEC analysis showed that cIL4-10 FP preparations mainly consisted of non-covalently linked dimers with a mass of ~70 kDa, as well as some monomers of ~35 kDa (Fig 3D). Both monomer and dimer have been demonstrated to be biologically active.

### Bioactivity of canine IL4-10 FP in vitro

**Fig 4:**
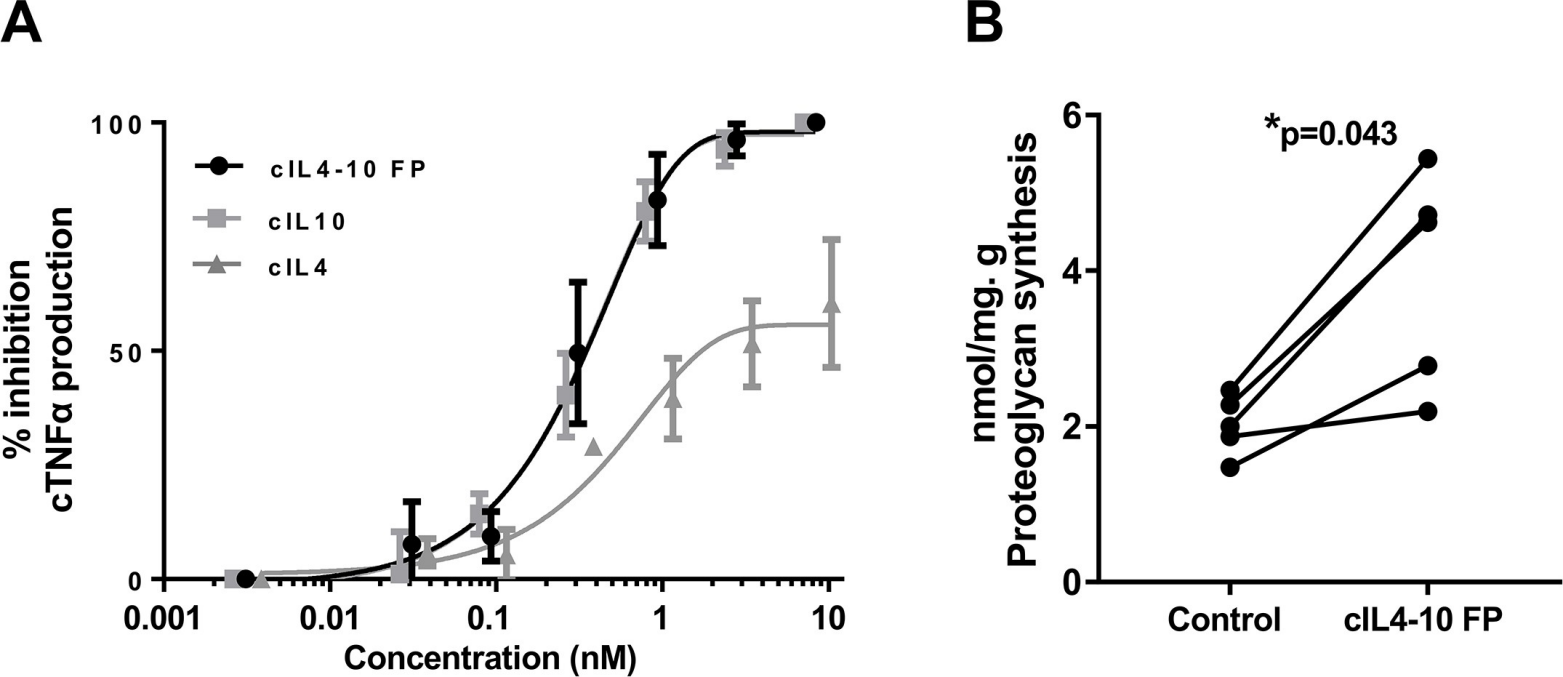
In vitro activity of cIL4-10 FP. A. Anti-inflammatory activity of cIL4-10 FP. Whole canine blood was stimulated with LPS with or without IL4-10 FP, IL10, or IL4 for 18 hours. Canine TNFα was measured using ELISA. B. Chondroprotective activity of cIL4-10 FP. Healthy canine cartilage explants were cultured in TNFα containing medium with or without cIL4-10 FP for four days. Proteoglycan synthesis rate was measured using liquid scintillation of ^35^SO_4_^2—^labelled GAGs. Values are expressed per dog and are the mean of eight tissue samples per dog. Mean proteoglycan synthesis rate was 2.0 and 4.0 for controls and cIL4-10 FP treated conditions, respectively.

The bioactivity of cIL4-10 FP was evaluated *in vitro* by its ability to diminish TNFα production in an LPS-stimulated canine whole blood culture. cIL4-10 FP dose-dependently inhibited TNFα production, with maximal inhibition at 3.0 nM. Bioactivity of the canine IL4-10 FP was comparable to that of canine IL-10, whilst IL-4 was less efficient (Fig 4A).

To test the chondroprotective activity of cIL4-10 FP, PG synthesis in TNFα-stimulated canine cartilage explants was evaluated *in vitro*. cIL4-10 FP statistically significantly improved PG synthesis in TNFα compromised cartilage explants as compared to untreated, TNFα compromised cartilage explants (4.0 (95%CI: 2.2–5.7) vs 2.0 (95%CI: 1.5–2.5), p = 0.043, Fig 4B).

### Canine IL4-10 FP in the canine Groove model of OA

#### Immunogenicity

**Fig 5:**
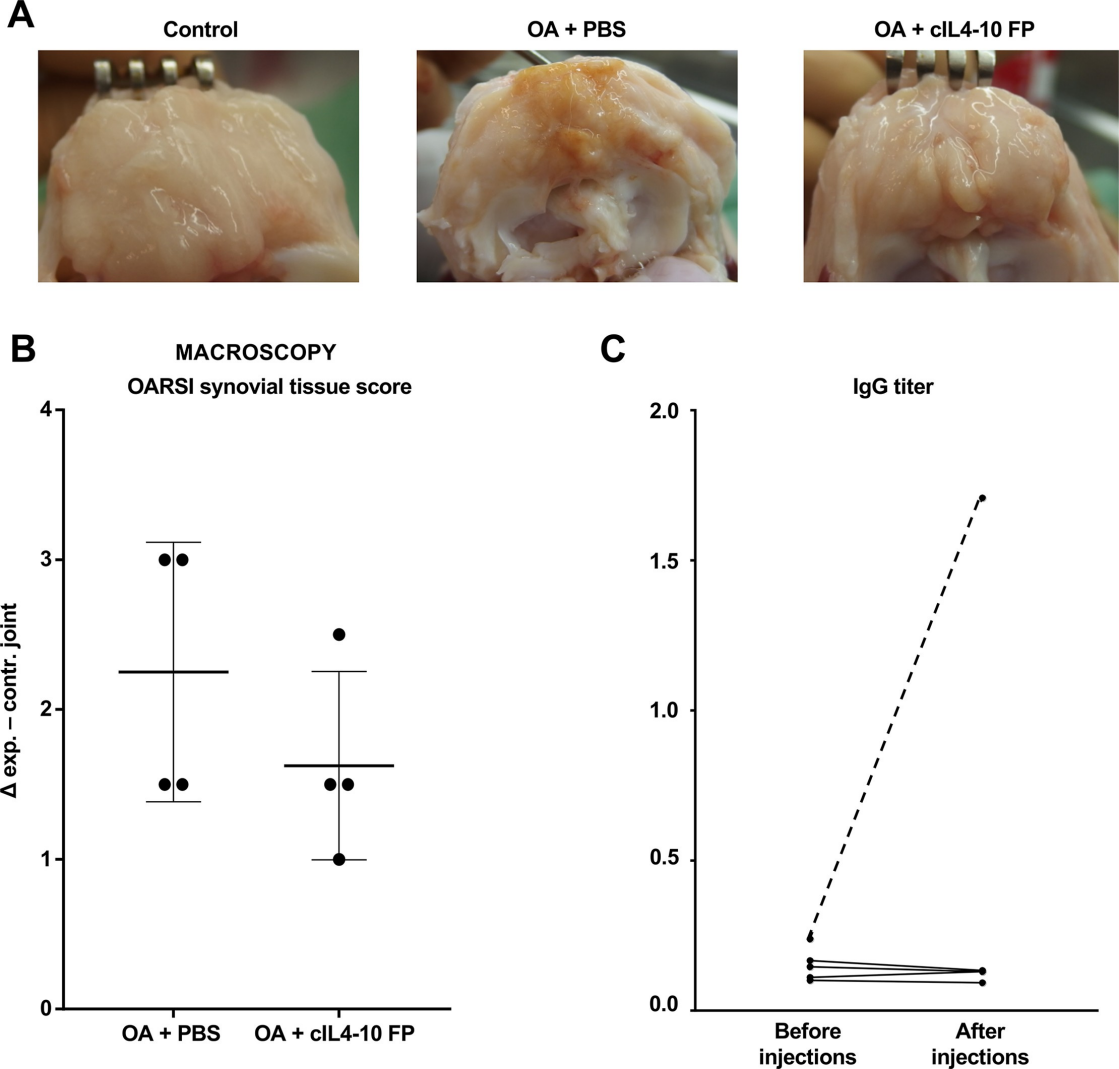
Immunogenicity of cIL4-10 FP in the canine Groove model of OA after injections with PBS or cIL4-10 FP. A. Macroscopic pictures of synovial fat pad of control, PBS injected and cIL4-10 FP injected knees. B. Differences in macroscopic synovial inflammation (OARSI score) between experimental and contralateral joint, representing a change from baseline to end of the study for the experimental joint. Values are expressed per dog (dots) and as median ± IQR (bars). Mean macroscopic OARSI scores in contralateral joints were 0.5 (95%CI: -0.2–1.2) and 0.4 (95%CI: -0.4–1.1) in PBS group and cIL4-10 FP group, respectively. Mean macroscopic OARSI scores in experimental joints were 2.8 (95%CI: 0.9–4.6) and 2.0 (95%CI: 1.4–2.7) in PBS group and cIL4-10 FP group, respectively. C. IgG titers in serum of cIL4-10 FP injected dogs (continuous line) and, as a positive control, a hIL4-10FP injected dog (dotted line).

After ten injections of cIL4-10 FP, OD450 values of serum in the ELISA for IgG antibodies against cIL4-10 FP were comparable to values obtained with baseline samples, showing that ten repeated i.a. injections with species-specific protein did not lead to antibody formation against cIL4-10 FP (Fig 5C). Intermediate time points and neutralizing effects were not evaluated any further.

#### Synovial inflammation

**Fig 6:**
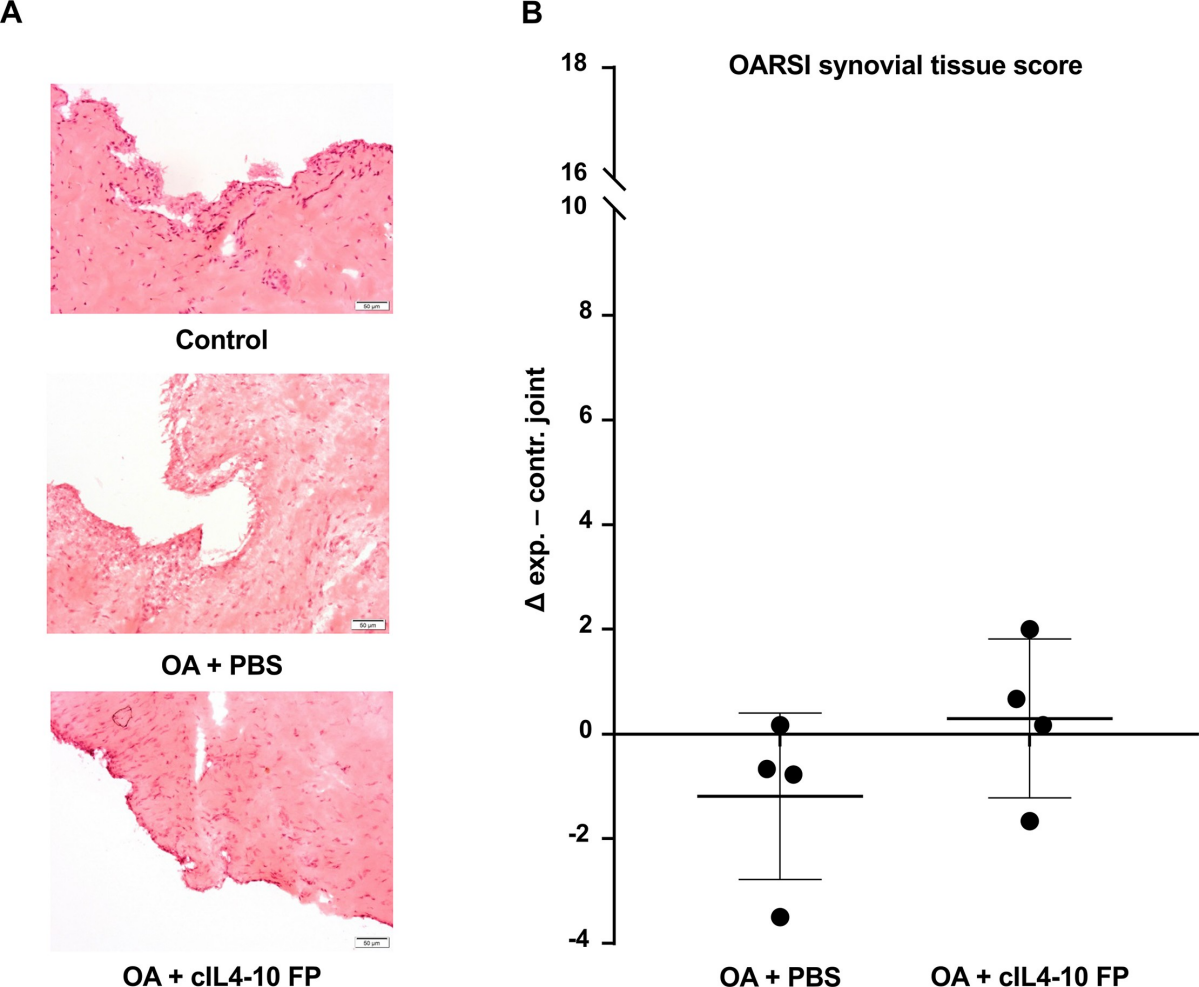
Histologic synovial inflammation in the canine Groove model of OA after injections with PBS or cIL4-10 FP. A. Representative images of Haematoxylin-eosin stained synovial tissue sections of control (without Groove surgery), PBS injected and cIL4-10 FP injected knees. Samples were collected at the end of the experiment; 16 weeks after Groove surgery and after ten weekly i.a. injections. B. Differences in histologic synovial inflammation (OARSI score) between experimental and contralateral joint, representing a change from baseline to end of the study for the experimental joint. Values are expressed per dog (dots) and as median ± IQR (bars). Mean histologic OARSI scores in contralateral joints were 3.0 (95%CI: 1.7–4.3) and 1.3 (95%CI: -0.1–2.6) in PBS group and cIL4-10 FP group, respectively. Mean histologic OARSI scores in experimental joints were 1.8 (95%CI: -0.8–4.5) and 1.5 (95%CI: -0.8–3.8) in PBS group and cIL4-10 FP group, respectively.

The Groove model is characterized by minimal signs of synovial inflammation[30, 32]. Macroscopic and histologic OARSI scoring of synovial inflammation showed indeed only minimal inflammation (Fig 5A representative macroscopic images and Fig 5B scores of the individual animals). As such analyzing anti-inflammatory activity was limited to the canine *in vitro* assays. Mean changes in histologic inflammation grade were -1.2 (95%CI: -3.7–1.3) and 0.3 (95%CI: -2.1–2.7) out of 18 for PBS injected dogs and cIL4-10 FP injected dogs, respectively (Fig 6). None of these changes were statistically significant (Table 1).

**Table 1:** Mean values of 4 dogs with p-values for all parameters of synovial inflammation and cartilage damage.

Parameter	PBS			cIL4-10 FP			
	Contr.	Exp.	Δ Exp.-Contr.	Contr.	Exp.	Δ Exp.-Contr.	p-value
**Force plate analysis**							
Vertical peak force (Fz) Braking force (Fy+) Propulsive force (Fy-)							**0.002** **0.010** 0.067
**Synovial inflammation**							
Macroscopy	0.5 (-0.2–1.2)	2.8 (0.9–4.6)	2.3 (0.9–3.6)	0.4 (-0.4–1.1)	2.0 (1.4–2.7)	1.6 (0.6–2.6)	0.343
Histology	3.0 (1.7–4.3)	1.8 (-0.8–4.5)	-1.2 (-3.7–1.3)	1.3 (-0.1–2.6)	1.5 (-0.8–3.8)	0.3 (-2.1–2.7)	0.886
**Cartilage damage**							
Macroscopy	0.5 (0.2–0.8)	1.8 (0.1–3.4)	1.3 (-0.1–2.6)	0.4 (0.1–0.6)	1.6 (1.2–1.9)	1.2 (0.6–1.8)	0.886
Histology - Cartilage structure - Chondrocyte pathology - PG staining	4.2 (1.2–7.2) 1.3 (-0.4–2.5) 0.5 (-0.2–1.1) 2.5 (1.2–3.8)	9.2 (5.7–12.7) 3.8 (3.0–4.5) 2.0 (-0.7–4.7) 3.5 (2.2–4.8)	5.1 (-1.3–11.4) 2.5 (0.6–4.4) 1.5 (-1.7–4.8) 1.0 (-1.3–3.3)	6.0 (1.7–10.3) 1.4 (0.0–2.8) 2.3 (-0.3–4.8) 2.3 (1.2–3.7)	7.2 (2.8–11.5) 2.1 (0.4–3.8) 1.9 (-0.6–4.4) 2.6 (1.9–3.3)	1.2 (-4.1–6.4) 0.7 (-0.6–2.0) -0.4 (-4.5–3.6) 0.3 (-1.2–1.8)	0.343 **0.057** 0.686 0.486
**Cartilage biochemistry**							
- PG synthesis - Release of newly formed PGs - PG content - Release of PGs	3.5 (-0.2–7.2) 79.7 (61.6–97.7) 37.8 (20.4–55.2) 10.5 (9.6–11.4)	1.4 (0.1–2.7) 92.0 (84.4–99.6) 27.8 (24.6–31.0) 12.0 (10.7–13.5)	-2.1 (-5.4–1.2) 12.4 (-10.8–35.6) -10.0 (-25.3–5.3) 1.6 (-0.4–3.5)	6.1 (-1.7–13.9) 75.7 (50.2–101.2) 30.7 (27.3–34.1) 12.6 (12.0–13.2)	3.5 (-3.4–10.3) 89.2 (74.4–104.0) 34.1 (28.3–39.9) 13.8 (10.9–16.7)	-2.7 (-9.3–4.0) 13.5 (-6.6–33.6) 3.4 (-2.6–9.4) 1.2 (-1.1–3.6)	0.686 0.886 **0.029** 0.886

Values are expressed as a mean of four dogs (95% CI). P-values represent results of Mann-Whitney U tests to compare changes within the group (Δexp.–contr.) between both groups.

#### Force plate analysis

**Fig 7:**
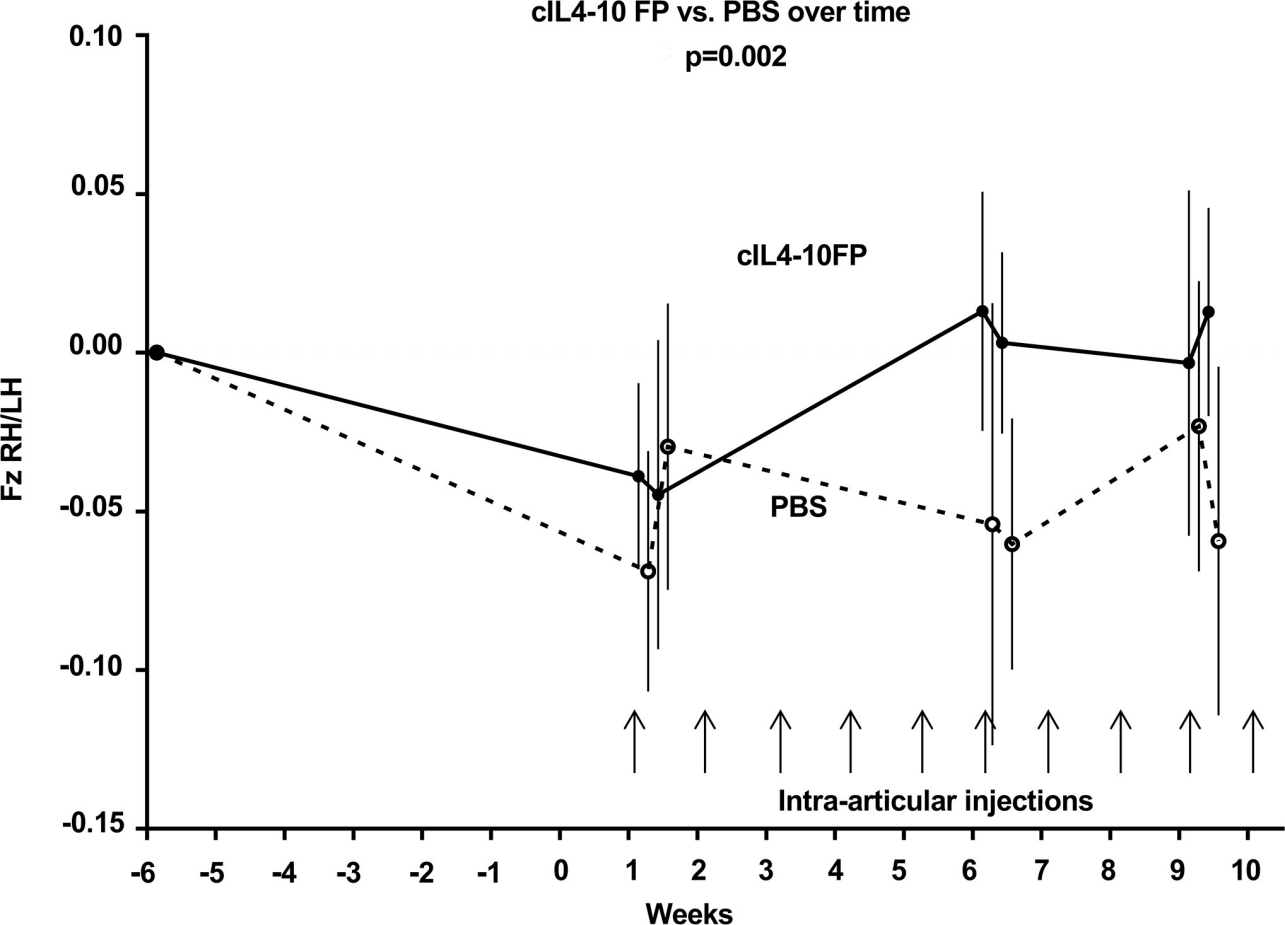
Analgesic effects of cIL4-10 FP in the canine Groove model of OA. Six weeks after Groove surgery dogs received weekly i.a. injections with cIL4-10 FP or PBS for ten weeks. Objective force plate analysis was used to determine joint loading as a proxy for pain. After OA induction, Fz RH/LH was clearly reduced. After treatment the Fz RH/LH statistically significant increased in the cIL4-10 FP treated group (continuous line, n = 4) compared to the PBS injected group (dotted line, n = 4); p = 0.002 over the whole time span. Values are expressed as a mean of four dogs ± SEM.

Groove surgery induced a reduction in stance (Fz) and propulsive force (Fy+) in both groups. After 10 i.a. injections with cIL4-10 FP Fz and Fy+ were restored to pre OA values. Overall, cIL4-10 FP increased Fz and Fy+ 24 hours after i.a. injections compared to PBS injected dogs (p = 0.002 and p = 0.01 for Fz and Fy+, respectively, Fig 7). Similarly, the breaking force (Fy-) trended to a decrease after surgery and a recovery after i.a. cIL4-10 FP injections, although not statically significant.

#### Cartilage damage

**Fig 8:**
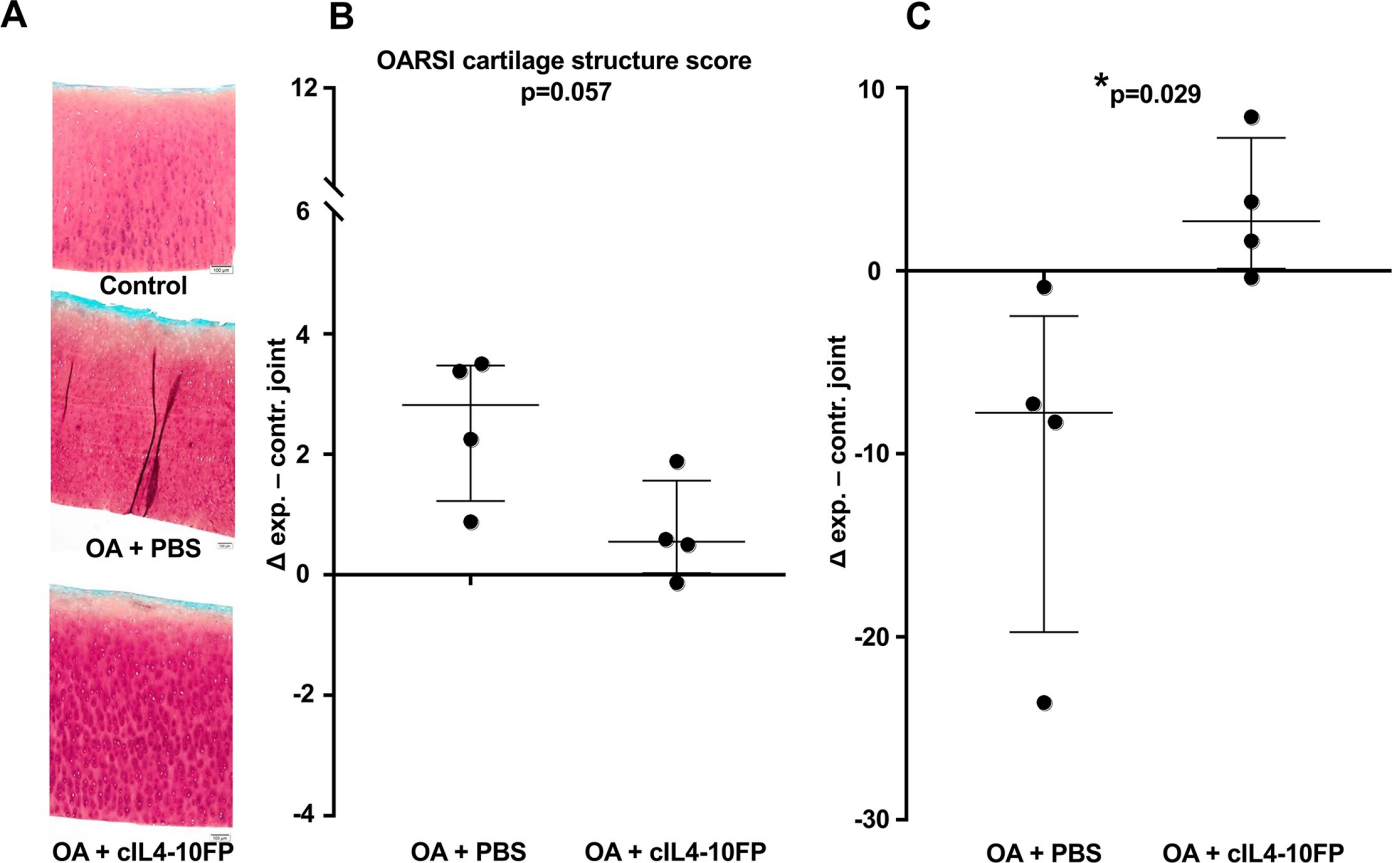
Cartilage damage in the canine Groove model of OA after injections with PBS or cIL4-10 FP. A. Representative images of Safranin-O stained tibial cartilage section of control (without Groove surgery), PBS injected and cIL4-10 FP injected knees. Samples were collected at the end of the experiment; 16 weeks after Groove surgery and after ten weekly i.a. injections. B. Differences in tibial cartilage structure (part of the OARSI score) between experimental and contralateral joint, representing a change from baseline to end of the study for the experimental joint. Values are expressed per dog (dots) and as median ± IQR (bars). Mean scores in contralateral joints were 1.3 (95%CI: -0.4–2.5) and 1.4 (95%CI: 0.0–2.8) in PBS group and cIL4-10 FP group, respectively. Mean histologic OARSI scores in experimental joints were 3.8 (95%CI: 3.0–4.5) and 2.1 (95%CI: 0.4–3.8) in PBS group and cIL4-10 FP group, respectively. C. Differences in proteoglycan content in tibia of injected knees. Values are expressed per dog (dots) and as median ± IQR (bars). Mean proteoglycan content in contralateral joints were 37.8 (95%CI: 20.4–55.2) and 30.7 (95%CI: 27.3–34.1) in PBS group and cIL4-10 FP group, respectively. Mean proteoglycan content in experimental joint were 27.8 (95%CI: 24.6–31.0) and 34.1 (95%CI: 28.3–39.9) in PBS group and cIL4-10 FP group, respectively.

Evaluation of Saf O stained cartilage sections showed a small increase in OARSI cartilage structural damage score in experimental (OA) knees compared to contralateral control knees (2.5 (95%CI: 0.6–4.4) points out of 12 points) in the PBS treated group, which was normalized in the cIL4-10 FP treated group (0.7 (95%CI: -0.6–2.0) points), at borderline significance (p = 0.057, Fig 8B). The other subscales of the OARSI score, chondrocyte pathology and proteoglycan staining, as well as total OARSI score, showed similar trends, although not statistically significant (Table 1).

The protective effect on cartilage was corroborated by PG content. This PG content was decreased in the OA joints injected with PBS (-10.0 (95%CI: -25.3–5.3) mg/g; p = 0.068) whereas in the cIL4-10 FP injected OA joints there was no difference in PG content compared to the contralateral control joint (+3.4 (95%CI: -2.6–9.4) mg/g; p = 0.144). cIL4-10 FP significantly normalized the reduced PG content; p = 0.029, compared to the PBS treated group (Fig 8C). Changes in PG synthesis, release, and retention did not statistically significant differ between both treatment groups although differences were mostly in favor of the cIL4-10 FP group (Table 1).

## Discussion

In a previous explorative *in vivo* study, no antibody formation was observed after two repeated injections of human IL4-10 FP in dogs[28]. This encouraged us to test the efficacy of multiple repeated injections of human IL4-10 FP in this canine model. However, ten weekly, i.a. injections with human IL4-10 FP induced significant IL4-10 FP neutralizing antibody formation that prevented evaluation of the effects of human IL4-10 FP. Although apparently obvious, this demonstrates that testing a human protein for longer periods in an animal model may induce unwanted antibody formation that may prevent therapeutic efficacy.

Therefore, in this study we designed a canine variant of the IL4-10 FP and tested this for its DMOAD activity in the *in vivo* canine Groove model.

A clear limitation of this study is the number of dogs treated with the cIL4-10 FP and, as a result, the lack of power. Due to ethical approval and budget reasons it was not possible to extend the number of dogs per group to the originally intended n = 8. The study also lacks a healthy control group. The use of the contralateral knee as an internal control is reported to be valid[31, 32] but has its limitations as altered loading or systemic alterations by development of the OA as well as the treatment may have been of influence.

Nevertheless, the value of using a large animal model with the financial and ethical limitations but significantly higher translational value to the human situation may be appreciated.

To ensure comparable pharmacological activity, the design of the canine IL4-10 FP, was similar to that of human IL4-10 FP and the *in vitro* bioactivity was evaluated in the same type of species-specific assays. Either fusion protein consisted of full length species-specific IL4 and IL10 sequences, connected with the same flexible glycine-serine-rich linker sequence. Indeed, cIL4-10 FP showed anti-inflammatory and chondroprotective activity in canine *in vitro* models, similar to the activity of hIL4-10 FP in human *in vitro* models[27, 28, 35]. In addition, cIL4-10 FP showed analgesic and chondroprotective effects in a *in vivo* canine model of OA, although the second part of the study was limited to four dogs per group. Importantly, canine IL4-10 FP did not induce antibody formation upon multiple i.a. injections in this canine model. The human variant of the IL4-10 FP has been demonstrated to be stable under different conditions and not delink. As such the delinking of the canine variant is not anticipated, due to its same construction, although not studied in detail.

According to previous findings, in this OA model only minimal synovial inflammation is observed in PBS injected knees, as well as in cIL4-10 FP injected knees, underlining the non-immunogenic nature of the species-specific IL4-10 FP. The low grade synovial inflammation in this model was an advantage in demonstrating potential i.a. immunogenic responses as well as inflammation independent chondroprotective effects, but more or less ruled out the evaluation of the anti-inflammatory activity of the cIL4-10 FP. Nevertheless, *in vitro* cIL4-10 FP had anti-inflammatory properties, shown by the ability to reduce LPS-induced TNFα production in canine whole blood. Moreover, hIL4-10 FP lowered the release of inflammatory cytokines from cartilage and synovial tissue, and decreased cartilage destructive properties of inflamed synovial tissue[27, 28, 35].

In addition to the potential anti-inflammatory effects, we here demonstrated that cIL4-10 FP is also chondroprotective *in vivo*. These findings are in line with the *in vitro* chondroprotective effect of cIl4-10 FP, normalizing the hampered PG synthesis rate of compromised OA canine cartilage. Moreover, hIL4-10 FP normalizes PG release and PG synthesis, as well as PG content in human *in vitro* OA cartilage experiments[28, 35].

In the present study, joint loading was used as a proxy for OA pain, induced by Groove surgery. A reduction in FPA parameters represent an objective outcome measure for evaluation of lameness, and to some extent of the magnitude of pain. However these measures do not mean that it tells us exactly how much pain dogs experience or, translating it to the human situation, how it affects quality of life[36]. It might be possible that dogs adapt their joint loading over time despite persisting pain. Irrespectively, we demonstrated a significant improvement in the OA induced reduction in joint loading over the injection period. These data corroborate our previous finding that two i.a. injections with hIL4-10 FP improved joint loading in the canine Groove model[28] prior to established systemic and local immunogenic response due to a species-mismatch. Overall these findings indicate that IL4-10 FP has analgesic properties. Indeed, the IL4-10 FP also inhibited pain in an *in vivo* rat Groove model[37] and two mouse models, confirming the analgesic capacity of the IL4-10 FP[27, 28, 38].

Altogether, the above-mentioned anti-inflammatory, chondroprotective, and analgesic properties clearly warrant further research to develop the IL4-10 FP as a DMOAD. In treatment of the larger joints, intra-articular treatment may be preferred above systemic treatment since it needs less (expensive) drug in comparison to systemic application, has a lower risk of systemic side effects, and targets the non-vascularized articular cartilage directly. This would favor patients and healthcare systems. However, application in generalized OA including more and smaller joints will be a challenge. As such, treatment at first focusses on local administration in the larger joints. However, a few issues need to be addressed before it can be brought into a clinical trial. Firstly, injecting patients every week is not feasible in clinical care. To overcome this problem, it is essential to find a way to enhance bioavailability in the joint. Currently, different molecule characteristics and slow release systems are being tested. Secondly, phenotypic differences between OA patients may warrant phenotypic treatment approaches (personalized medicine). IL4-10 FP consists of two anti-inflammatory cytokines, therefore future *in vivo* studies are needed to evaluate whether or not an inflammatory phenotype is the most responsive to this FP.

In conclusion, human IL4-10 FP is immunogenic in dogs upon multiple repeated i.a. injections. Canine IL4-10 FP can be produced and diminishes canine blood cell inflammatory activity and improves PG synthesis of canine cartilage. In addition, the canine IL4-10 FP shows promising DMOAD activity *in vivo* in the canine Groove model. Further research to develop the IL4-10 FP as a DMOAD is clearly warranted.

## Supporting information

S1 ARRIVE checklist (PDF)

S1 Safranin O Results OARSI scoring. (DOCX)
