# Supplementary material for: Canine IL4-10 fusion protein provides disease modifying activity in a canine model of OA; an exploratory study
Source: PLoS One. 2019 Jul 11;14(7):e0219587. doi: 10.1371/journal.pone.0219587 (PMC6622543; doi:10.1371/journal.pone.0219587)
Supplement: S1 Safranin O Results — (DOCX) [file pone.0219587.s002.docx]

**S2: Safranin O results – OARSI scoring (Cook et al. 2010)**

Cartilage section were scored by two blinded observers for:

1. Cartilage structure (0-12)
2. Chondrocyte pathology (0-12)
3. Proteoglycan staining (0-12)

**PBS group**

| **Dog** | **Site** | **Sample** | **Observer A** | | | **Observer B** | | | **Mean total score** |
| --- | --- | --- | --- | --- | --- | --- | --- | --- | --- |
|  | | | A | B | C | A | B | C |  |
| 1 | Left  (Control) | 1 | 1 | 0 | 0 | 2 | 0 | 0 | 2.13 |
|  |  | 2 | 0 | 0 | 3 | 0 | 0 | 3 |  |
|  |  | 3 | 0 | 0 | 0 | 0 | 0 | 0 |  |
|  |  | 4 | 1 | 0 | 3 | 1 | 0 | 3 |  |
|  | Right  (Experimental) | 5 | - | - | - | - | - | - | 12.17 |
|  |  | 6 | 3 | 8 | 3 | 3 | 9 | 3 |  |
|  |  | 7 | 4 | 3 | 4 | 4 | 3 | 4 |  |
|  |  | 8 | 5 | 2 | 4 | 5 | 2 | 4 |  |
| 2 | Left  (Control) | 1 | - | - | - | - | - | - | 6 |
|  |  | 2 | - | - | - | - | - | - |  |
|  |  | 3 | 1 | 0 | 3 | 1 | 0 | 3 |  |
|  |  | 4 | 2 | 2 | 3 | 3 | 2 | 4 |  |
|  | Right  (Experimental) | 5 | 4 | 2 | 3 | 4 | 2 | 2 | 8.25 |
|  |  | 6 | - | - | - | - | - | - |  |
|  |  | 7 | 3 | 0 | 4 | 5 | 0 | 4 |  |
|  |  | 8 | - | - | - | - | - | - |  |
| 3 | Left  (Control) | 1 | - | - | - | - | - | - | 3 |
|  |  | 2 | - | - | - | - | - | - |  |
|  |  | 3 | 1 | 1 | 1 | 1 | 0 | 1 |  |
|  |  | 4 | 0 | 1 | 3 | 0 | 0 | 3 |  |
|  | Right  (Experimental) | 5 | - | - | - | - | - | - | 9.5 |
|  |  | 6 | 1 | 0 | 2 | 2 | 0 | 2 |  |
|  |  | 7 | - | - | - | - | - | - |  |
|  |  | 8 | 6 | 2 | 6 | 7 | 2 | 8 |  |
| 4 | Left  (Control) | 1 | 3 | 0 | 3 | 4 | 0 | 3 | 5.5 |
|  |  | 2 | 2 | 1 | 3 | 2 | 0 | 3 |  |
|  |  | 3 | 2 | 1 | 3 | 4 | 0 | 3 |  |
|  |  | 4 | 0 | 1 | 3 | 0 | 0 | 3 |  |
|  | Right  (Experimental) | 5 | 0 | 0 | 3 | 2 | 0 | 3 | 7 |
|  |  | 6 | 0 | 1 | 2 | 1 | 0 | 1 |  |
|  |  | 7 | - | - | - | - | - | - |  |
|  |  | 8 | 9 | 4 | 3 | 6 | 4 | 3 |  |

**cIL4-10 FP group**

| **Dog** | **Site** | **Sample** | **Observer A** | | | **Observer B** | | | **Mean total score** |
| --- | --- | --- | --- | --- | --- | --- | --- | --- | --- |
|  | | | A | B | C | A | B | C |  |
| 1 | Left  (Control) | 1 | - | - | - | - | - | - | 6.5 |
|  |  | 2 | 2 | 2 | 3 | 3 | 2 | 3 |  |
|  |  | 3 | 2 | 3 | 3 | 2 | 3 | 3 |  |
|  |  | 4 | 0 | 1 | 3 | 0 | 1 | 3 |  |
|  | Right  (Experimental) | 5 | 6 | 4 | 3 | 6 | 4 | 3 | 10 |
|  |  | 6 | 3 | 3 | 3 | 3 | 3 | 3 |  |
|  |  | 7 | 3 | 3 | 4 | 3 | 2 | 4 |  |
|  |  | 8 | 1 | 4 | 1 | 2 | 8 | 1 |  |
| 2 | Left  (Control) | 1 | - | - | - | - | - | - | 7.75 |
|  |  | 2 | - | - | - | - | - | - |  |
|  |  | 3 | 0 | 4 | 3 | 1 | 4 | 2 |  |
|  |  | 4 | 3 | 3 | 3 | 3 | 2 | 3 |  |
|  | Right  (Experimental) | 5 | - | - | - | - | - | - | 4.25 |
|  |  | 6 | - | - | - | - | - | - |  |
|  |  | 7 | 1 | 0 | 1 | 1 | 0 | 2 |  |
|  |  | 8 | 3 | 0 | 2 | 4 | 0 | 3 |  |
| 3 | Left  (Control) | 1 | - | - | - | - | - | - | 2 |
|  |  | 2 | 0 | 1 | 3 | 0 | 0 | 3 |  |
|  |  | 3 | 0 | 0 | 2 | 1 | 0 | 2 |  |
|  |  | 4 | 0 | 0 | 0 | 0 | 0 | 0 |  |
|  | Right  (Experimental) | 5 | - | - | - | - | - | - | 5.5 |
|  |  | 6 | 1 | 1 | 3 | 1 | 1 | 3 |  |
|  |  | 7 | 0 | 3 | 3 | 1 | 2 | 3 |  |
|  |  | 8 | - | - | - | - | - | - |  |
| 4 | Left  (Control) | 1 | - | - | - | - | - | - | 7.75 |
|  |  | 2 | 2 | 1 | 2 | 2 | 4 | 1 |  |
|  |  | 3 | 3 | 4 | 2 | 2 | 6 | 2 |  |
|  |  | 4 | - | - | - | - | - | - |  |
|  | Right  (Experimental) | 5 | - | - | - | - | - | - | 8.92* |
|  |  | 6 | - | - | - | - | - | - |  |
|  |  | 7 | - | - | - | - | - | - |  |
|  |  | 8 | - | - | - | - | - | - |  |

- Score was calculated by taking the mean change of the cIL4-10 FP group (=1.17) and add this to the left/control knee
